# Sensitivity of Microscopy Compared to Molecular Diagnosis of *P. Falciparum:* Implications on Malaria Treatment in Epidemic Areas in Kenya

**DOI:** 10.4314/ajid.v5i1.66504

**Published:** 2011

**Authors:** Laura Nyawira Wangai, Muriira Geoffrey Karau, Paul Nthakanio Njiruh, Omar Sabah, Francis Thuo Kimani, Gabriel Magoma, Njagi Kiambo

**Affiliations:** 1 Institute of Tropical Medicine and Infectious Diseases (ITROMID), P.O. Box 54840-00200 Nairobi, Kenya; 2 Jomo Kenyatta University of Agriculture and Technology (JKUAT), Biochemistry Department, P.O. Box 62000-00200, Nairobi, Kenya; 3 Kenya Bureau of Standards, Department of Research and Development, P. O Box 54974-00200, Nairobi, Kenya; 4 Kenya Polytechnic University College, P. O Box 52428-00200, Nairobi, Kenya; 5 Kenya Medical Research Institute, Centre for Biotechnology Research and Development (KEMRI-CBRD), P. O Box 54840-00200 Nairobi, Kenya; 6 Division of Malaria Control, Ministry of Health, Kenya

**Keywords:** *Plasmodium falciparum*, diagnosis, epidemics, microscopy, polymerase chain reaction

## Abstract

Detection of *Plasmodium* species by microscopy has been the gold standard for diagnosis of malaria for more than a century. Despite the fact that there is a significant decline in the number of positive cases reported from microscopy, antimalarial drugs prescriptions are on continuous increase as patients present with symptoms of malaria. This makes it difficult to establish accuracy, sensitivity and specificity of light microscopy in diagnosis of malaria in epidemic areas. This study was designed to compare microscopy with polymerase chain reaction as diagnostic methods for malaria in three epidemic areas in Kenya. A total of 356 patients presenting with malaria symptoms were diagnosed by microscopy and dried blood filter paper spots were collected from patient in Kisii, West Pokot and Narok districts. *Plasmodium falciparum* DNA was extracted from the dried blood filter samples. Primers specific for the *Plasmodium Species* were designed and used in a two step amplification of the *Pfmdr* gene. The PCR products were analyzed in ethidium bromide stained 1.5% agarose gel. It was found that 72 out of 350 specimens diagnosed as negative were positive for *P. falciparum* by nested PCR, while 6 which were microscopy positive were confirmed so by nested PCR. This study demonstrates that there is a high level of misdiagnosis which may either lead to denial for deserved treatment or undeserved treatment. Nested PCR detection of malaria parasites is a very useful complement to microscopy although it is expensive and takes long time. Additionally, smear negative patients suspected to have malaria should be subjected to PCR diagnosis to improve rational drug use. The economic burden of misdiagnosis and mistreatment of malaria outweighs that of PCR diagnosis, hence this diagnostic mode could be tenable in the long run even in rural areas.

## Introduction

Malaria, a tropical disease caused by protozoan parasites of the genus *Plasmodium* is one of the most important infectious diseases in the world (Breman, 2001). Malaria kills over a million people each year, with as many as 300–500 million people being infected, with extremely high fatality rates among young children below 5 years of age (WHO, 2005). Furthermore, anti-malarial drug resistance has become as one of the greatest challenges against malaria control, drug-resistance to Chloroquine and more recently quinine was responsible in the spread of malaria to new areas and occurrence of malaria in areas where the disease has been eradicated. Proper diagnosis of *Plasmodium falciparum* has played an important role in the occurrence, severity and management of malaria epidemics. Sensitive, accurate and specific methods of diagnosis are important in proper treatment.

Light microscopy of thick and thin stained blood smears remains the gold standard method for diagnosing malaria (Moody and Chiodini, 2000). Thick smears are 20–40 times more sensitive than thin smears for screening of *Plasmodium* parasites, with a detection limit of 10–50 trophozoites/µl (Trampuz et al., 2003). Thin smears allow one to identify malaria species (including the diagnosis of mixed infections), quantify parasitemia, and assess for the presence of schizonts, gametocytes, and malarial pigment in neutrophils and monocytes (Trampuz et al., 2003). The diagnostic accuracy relies on the quality of the blood smear and experience of laboratory personnel. Although examination of the thick and thin blood smear is the ‘gold standard’ for diagnosing malaria, important advances have been made in diagnostic testing, including fluorescence microscopy of parasite nuclei stained with acridine orange, rapid dipstick immunoassay, and polymerase chain reaction (PCR) assays. Sensitivity and specificity of some of these methods exceed by far those of the thin and thick smear. Diagnosis based on polymerase chain reaction for species-specific *Plasmodium* genome are the most accurate, sensitive and specific compared to diagnostic methods, and are capable of detecting as few as 10 parasites/µl of blood (Hanschield, and Grobusch, 2002). WHO recommends that malaria be confirmed by parasite-based diagnosis before giving treatment (WHO, 2005). In developing world where malaria is highly prevalent, the resources to aid in proper diagnosis are lacking (Rafael et al., 2006). This lack of resources to aid proper and accurate diagnosis of *P. falciparum* has lead to improper administration of anti-malarials.

The present study compared the sensitivity, specificity and accuracy of microscopy with those of polymerase chain reaction (PCR) technique. This was prompted by a decline in the number of positive *Plasmodial* cases detected by microscopy, though clinical prescriptions for anti-malarial are on increase. This implies that treatment of malaria in epidemic areas should be carefully carried out following a proper diagnosis.

## Materials and Methods

This present study was conducted from months of October to December, 2007 during in door residual spraying campaigns in the districts of Kisii, Narok and West Pokot in Western Province of Kenya. The malaria vectors in these areas are *A. gambiae, A. funestus*, and *A.arabiensis* (Castro et al., 2004). The malaria transmission in these districts is seasonal and epidemic. Generally, the rainfall pattern is bimodal, with a long rainy season between March and May and a short rain season between October and December.

The study protocol was approved by the scientific steering committee and the ethical review committee of the Kenya Medical Research Institute (KEMRI), Kenya. Written and informed consent forms presented in native language and translated to the patients were obtained from adults and/ or parent or guardian of the children participating in the study. Blood samples were collected as dry blood spots on Whatman 3M filter paper from 129, 99 and 122 patients diagnosed as *P. falciparum* negative by microscopy from Kisii, Narok and West Pokot districts, respectively.

### DNA Extraction

DNA was extracted by Chelex method according to Snounou et al., 1993 with few modifications. Briefly, 4mm^2^ piece of filter paper with blood spot was cut with a sterile scalpel blade and incubated in 0.5% saponin in 1 × PBS overnight at 4° C. The brown solution was removed and replaced with 1 × PBS and then incubated for 20 mins. The solution was removed and 100µl of DNAse free water was added followed by 50µl of 20% Chelex. The tubes were placed into a heated block and voltexed every two mins. This was repeated up to 5 times. The solution was centrifuged and the supernatant carefully separated. The supernatant contains DNA and 30µl aliquots were taken into eppendorf tubes and stored at −20° C for PCR analysis.

### Screening *P. falciparum* microscopy negative samples by nested PCR

Nested PCR was carried out targeting *Pfmdr1* gene on MJ Thermocycler™. The following were the primers designed for outer PCR with primer 3.0 Software. Primers, MDR/A1 (TGT TGA AAG ATG GGT AAA GAG CAG AAA GAG) and MDR/A3 (TAC TTT CTT ATT ACA TAT GAC ACC ACA AAC) for forward and reverse amplification respectively were designed by primer 3.0 software. The reaction mixture comprised of 2µl of template DNA, 2.5 mM magnesium chloride, 100nM dNTP, 100nm of each primer and Taq polymerase at 1unit/reaction to a total volume of 30µl. The cycling conditions were set at 94°C for initial denaturation for 3 minutes followed by denaturation at 94°Cfor 1 min, annealing at 45°C for 3 seconds and extension at72°C for 1 minute. These conditions were repeated for 40 cycles.

The following primer pair was used in nested PCR was for forward and reverse amplifications respectively; MDR/A2 (GTC AAA CGT GCA TTT TTT ATT AAT GAC CAT TTA) and MDR/A4 (AAA GAT GGT AAC CTC AGT ATC AAA GAA GAG). Nest 1 PCR products were thawed on ice and 5µl from each tube was transferred into labeled sterilized PCR tube. The reaction mixture for nested PCR for *Pfmdr*1 comprised of 1X PCR Buffer, 2.5mM magnesium chloride, 100nM dNTPs, 100nm of each primer MDR/A2 and MDR/A4 and Taq polymerase at 1unit/reaction to a total volume of 30µl. The thermal cycling conditions were, denaturation at 94 ° C for 30 seconds followed by annealing at 45 ° C for 1 minute and extension at 72 ° C for 1minute. These steps were repeated for 40 cycles and then followed by a final extension at 72 ° C for 3 mins before halting the reaction at 4°C.

### PCR products analysis by gel electrophoresis

The PCR products were confirmed and analysed on a1.5% agarose (Sigma) gel electrophoresis with 0.5µg/ml of ethidium bromide (Promega). The electrophoresis gel run for 30 minutes at 80 volts on a horizontal electrophoretic tank (Bio Rad) submerged with 1× TAE buffer. DNA amplification products were visualized under ultraviolet light against a 100 base pair marker (Roche) on a transilluminator (Vilber Lourmat) and the results documented using Polaroid® camera and Polaroid® instant films.

## Results and Discussions

A total of 356 patients with ages between 4 to over 50 years participated in this study. There were 132 patients from Kisii, 99 from Narok and 125 from West Pokot district. These patients were examined for malaria by thick blood film by an experienced technician in field stations. It was found that 350 patients were *P. falciparum* negative and 6 positive by microscopy. For the *P. falciparum* negative, 129, 99 and 122 patients were from Kisii, Narok and west Pokot, respectively.

Seventy two (72) making up 20.57% of cases diagnosed as negative by microscopy were found to be positive for *P. falciparum* with nested-PCR. The percentages of *P. falciparum* positive patients were, 20.9% (n=27) in Kisii, 7.1% (n=7) in Narok and 31.1% (n=38) in west Pokot.

### Microscopy versus PCR

**Figure 1:**
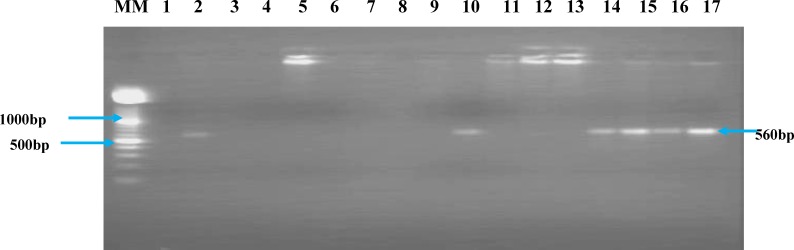
Representative gel showing nested PCR products on 1.5% agarose gel electrophoresis of *P. falciparum* negative blood filters determined by microscopy. MM is the molecular weight marker, Lane 1, represents the negative control, Lane 2, the positive control and lanes 3–17 are the *P. falciparum* negative samples by microscopy from the epidemic areas.

The accuracy, sensitivity and specificity of PCR were evaluated against those of microscopy. These values were calculated using InStat® statistical software, taking microscopy as the gold standard while PCR as the test method (www.graphpad.com/instat3/instat.htm). The sensitivity, specificity and accuracy of PCR on all the samples collected were 100%, 79% and 39.9% respectively. Kisii samples had a sensitivity of 100%, specificity 79% and accuracy of 39.7%. Narok samples had a specificity 92.9% and accuracy level of 39.9%. West Pokot had sensitivity of 100%, specificity of 68.9 % and accuracy of 34%. These results are shown in Table 1 and Figure 1. Using Chi-square P< 0.0001 value was obtained at 95% confidence interval.

**Table 1:** Shows the accuracy, sensitivity and specificity of PCR against microscopy for the samples collected from Kisii, Narok and west Pokot. These values were computed from www.graphpad.com/instat3/instat.htm taking microscopy as the gold standard

STUDY SITE	SENSITIVITY %	SPECIFICITY %	ACCURACY OF THE TEST %
KISII	100	79	39.7
NAROK	***	92.9	***
WEST POKOT	100	68.9	34

**Legend:** ***, no values for sensitivity and accuracy for the samples from Narok.

For the sensitivity and accuracy of samples from Narok, there were no values because there were no positive samples by microscopy giving an infinite value.

Diagnosis of malaria currently depends on the visualization of parasites by light microscopy of Giemsa-stained thick and thin blood smears. Although this method is cheap and simple it is labour intensive and requires a well trained personnel. Many studies have demonstrated that PCR method is more sensitive, accurate and specific compared to thick blood films diagnosis of *P. falciparum*. The detection of low *P. vivax* and *P. falciparum* parasitaemia by PCR, at levels undetectable by microscopy, has been reported (Snounou et al., 1993).

Rapid dipstick immunoassays detect species-specific circulating parasite antigens targeting either the histidine-rich protein-2 of *P. falciparum* or a parasite-specific lactate dehydrogenase (Moody, 2002). Although the dipstick tests may enhance diagnostic speed, microscopic examination remains mandatory in patients with suspected malaria, because occasionally these dipstick tests are negative in patients with high parasitemia, and their sensitivity below 100 parasites/µl is low (Moody, 2002).

The present study compared microscopy with nested PCR method of detection. It was found that results obtained by PCR method were superior to those obtained by microscopy. This is because 72 (20.57%) out of 350 microscopy negative samples were found to be positive by PCR and all microscopy-positive samples were confirmed as positive by PCR. Hence PCR would be preferred to microscopy for the confirmation of clinical suspicion of malaria. However, PCR method is too cumbersome, expensive and not available in the local set ups where there are limited resources (Hanscheid and Grobusch, 2002). With the spread of *P. falciparum* resistant to anti-malarial drugs in various provinces and the increasing difficulty in controlling malaria, it is important to diagnose and treat malaria accurately. Microscopic observation of parasites stained with Giemsa in thick smears is an inexpensive and simple method that is still used.

Several malaria infections from endemic countries are sub patent, with very low parasitemia, and our results also showed this has occurred in our study area (Hayder et al., 2005). Selection of drugs for treating malaria depends on species of *Plasmodia* detected in the blood of the patient. Delayed or missed diagnosis of *falciparum* malaria increases the risk of complicated or severe disease, which may be fatal, especially in non-immunes. Many isolates of *P. falciparum* are chloroquine resistant and thus would not be eradicated by the standard treatment for *P. vivax*. A missed diagnosis of *P. vivax* concurrent with *P. falciparum* is more problematic since these species could cause relapses, thereby compounding morbidity. Because of negative diagnosis by microscopy, untreated patients may be carriers of the *Plasmodia* parasites. These results suggest that, in malaria epidemic areas where there is transmission of *P. falciparum*, diagnosis of P. *falciparum* malaria by nested PCR is a very useful complement to microscopical examination. This is crucial in obtaining data on the incidences of each *Plasmodium* species and also for the follow-up of patients after specific treatment.

Microscopy has been found to have a great number of limitations. A superior and a more reliable diagnostic technique need to be put in place to enable proper treatment and control of malaria. The recent increase in population movement to and from areas of high malaria transmission intensity through domestic tourism, as well as migration due to conflicts and socioeconomic factors, has resulted in higher numbers of malaria cases, where a parallel increase of mortality, from 3.8 to 20%, has been mostly ascribed to late or incorrect diagnosis (Rubio et al., 1999). Despite treatment, between 1% and 4% of travelers who acquire *P. falciparum* malaria will die as a result of infection. This fatality rate increases to 20% or higher in patients who develop severe malaria, pregnant women or the elderly. Since 90% of people who contract malaria will not become ill until the level of parasitemia is really high (Kain et al., 1998). Sensitive routine laboratory techniques for rapid and accurate malaria are therefore desirable; first for diagnosis on admission, so as to initiate proper treatment and second during the follow up period in order to effectively manage and control malaria.

Although detection of *Plasmodium* parasite on Giemsa-stained blood smears by microscopy has been the reference standard for malaria diagnosis in laboratories for more than a century, it is an imperfect standard which depend mainly on the technical expertise and experience of the microscopist (Moody, 2002). The ability to maintain the required level of expertise in malaria diagnosis is a challenge particularly in periphery medical centers where the disease is not endemic (Drakeley et al., 2005). Microscopy is time consuming, can lead to misdiagnosis if the microscopist is inexperienced and /or when parasitemia is low (Hayder et al., 2005). Also, microscopy is not ideal for diagnosis of mixed infections.

Alternative techniques for laboratory diagnosis of malaria have been developed for use in both endemic and epidemic areas (Hawkes, and Kain, 2007). Serological diagnostic methods and new rapid diagnostic test (RDT) for detection of antigen e.g. Parasight F (Becton Dickinson), ICT Malaria Pf/Pv (ICT Diagnostic) and OptiMAL (Flow Inc.) have been in use for detection of antigens. Though they offer an advantage in that results can be obtained within half an hour by non skilled technicians, they are tempered by three limitations (Moody, 2002). RDT methods do not offer improved sensitivity over microscopy; the sensitivity decreases as parasitemia fall below 100parasites/µl; false positives are observed particularly after treatment, as the parasites antigens detected can remain in the circulation following parasite clearance. Finally, current RDT are either specific to *P. falciparum* or they cannot distinguish between the parasite species present. Several PCR assays for malaria diagnosis have also been developed, based on species-specific sequences of the parasite's 18S subunit rRNA gene (Snounou et al., 1993). This make PCR assays ideal over microscopy.

The economic cost of misdiagnosed or wrongly treated malaria cannot be compared with the cost of introducing PCR diagnosis. Delays in recognition and appropriate treatment of malaria increase morbidity and mortality (Kain et al., 1998). Thick smears are 20–40 times more sensitive than thin smears for screening of Plasmodium parasites, with a detection limit of 10–50 trophozoites/µl. Thin smears allow one to identify malaria species (including the diagnosis of mixed infections), quantify parasitemia, and assess for the presence of schizonts, gametocytes and malarial pigment in neutrophils and monocytes. The diagnostic accuracy relies on the quality of the blood smear and experience of laboratory personnel. Tests based on PCR for species-specific *Plasmodium* genome are more sensitive and specific than are other tests, detecting as few as 10 parasites/µl of blood (Hanscheid, and Grobusch, 2002). Based on these diagnosis disparities and capabilities, it is clear that most malaria deaths are caused by misdiagnosis which leads to mistreatment.

## Conclusion

In this study, an AmpliTaq-based PCR qualitative assay for the rapid detection and identification in clinical specimens of *P. falciparum* species of malaria parasite is described and evaluated using blood samples obtained from patients diagnosed by microscopy as negative. PCR-based methods have been demonstrated to be approximately 10-fold sensitive than microscopy. Putting into consideration the specificity and sensitivity of PCR-based assays, adoption of the technique as a routine diagnostic test- mainly aimed at patients presenting with mixed infection and febrile illness- will go a long way in eliminating cases of misdiagnosis and provide a window for early detection of malaria parasites which will enable the physician administer proper drugs and in good time. Again, the economic cost of misdiagnosis and mistreatment is over that of PCR diagnosis. This method should be adopted in rural settings to reduce mortality and morbidity caused by malaria due to poor diagnosis.

## Figures and Tables

**Figure 2:**
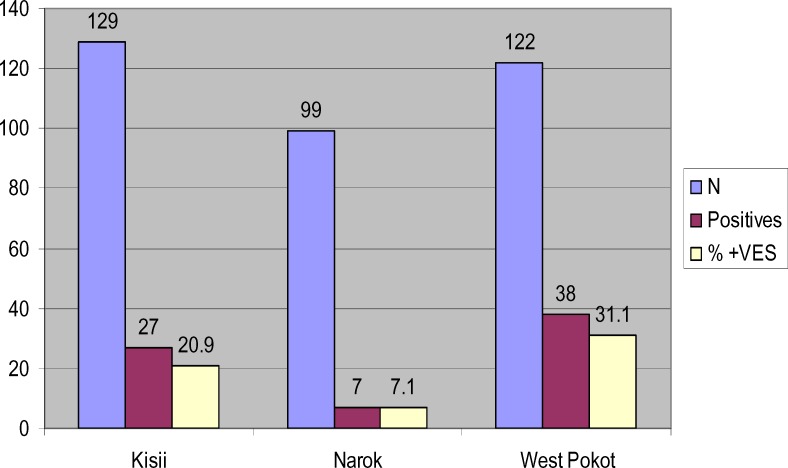
Bar graph showing the number of positive by nested PCR from the samples diagnosed as negative by microscopy and the percentages of the positive from each district studied
